# Efficacy of laser capture microdissection plus RT-PCR technique in analyzing gene expression levels in human gastric cancer and colon cancer

**DOI:** 10.1186/1471-2407-8-210

**Published:** 2008-07-25

**Authors:** Hiroshi Makino, Hiroyuki Uetake, Kathleen Danenberg, Peter V Danenberg, Kenichi Sugihara

**Affiliations:** 1 Department of Surgical Oncology, Tokyo Medical and Dental University, 1-5-45 Yushima, Bunkyo-ku, Tokyo 113-8519, Japan; 2 Department of Translational Oncology, Tokyo Medical and Dental University, 1-5-45 Yushima, Bunkyo-ku, Tokyo 113-8519, Japan; 3 Norris Comprehensive Cancer Center, University of Southern California, 1441 Eastlake Avenue, Los Angeles, CA 90033, USA

## Abstract

**Background:**

Thymidylate synthase, dihydropyrimidine dehydrogenase, thymidine phosphorylase, and orotate phosphoribosyltransferase gene expressions are reported to be valid predictive markers for 5-fluorouracil sensitivity to gastrointestinal cancer. For more reliable predictability, their expressions in cancer cells and stromal cells in the cancerous tissue (cancerous stroma) have been separately investigated using laser capture microdissection.

**Methods:**

Thymidylate synthase, dihydropyrimidine dehydrogenase, thymidine phosphorylase, and orotate phosphoribosyltransferase mRNA in cancer cells and cancerous stroma from samples of 47 gastric and 43 colon cancers were separately quantified by reverse transcription polymerase chain reaction after laser capture microdissection.

**Results:**

In both gastric and colon cancers, thymidylate synthase and orotate phosphoribosyltransferase mRNA expressions were higher (p < 0.0001, p <0.0001 respectively in gastric cancer and P = 0.0002, p < 0.0001 respectively in colon cancer) and dihydropyrimidine dehydrogenase mRNA expressions were lower in cancer cells than in cancerous stroma (P = 0.0136 in gastric cancer and p < 0.0001 in colon cancer). In contrast, thymidine phosphorylase mRNA was higher in cancer cells than in cancerous stroma in gastric cancer (p < 0.0001) and lower in cancer cells than in cancerous stroma in colon cancer (P = 0.0055).

**Conclusion:**

By using this method, we could estimate gene expressions separately in cancer cells and stromal cells from colon and gastric cancers, in spite of the amount of stromal tissue. Our method is thought to be useful for accurately evaluating intratumoral gene expressions.

## Background

Gastrointestinal cancers are major causes of cancer death throughout the world [1,2]. Recent advances of multimodal treatments have been improving the prognosis. Chemotherapy with combinations of new drugs including fluoropyrimidine, irrinotecan, and oxaliplatin, has greatly contributed to the prolonged prognosis [3-8].

5-Fluorouracil (5-FU) is a key drug in combination chemotherapy and an evaluation of the predictability of 5-FU sensitivity is important to exclude those patients who would experience adverse effects. Among the molecular markers of 5-FU activity, thymidylate synthase (TS), dihydropyrimidine dehydrogenase (DPD), orotate phosphoribosyltransferase (OPRT), and thymidine phosphorylase (TP) are reported to be highly predictive of 5-FU sensitivity [9-23]. 5-FU is catabolized to dihydrofluorouracil and inactivated by DPD. Thymidylate synthase is an essential DNA synthetic enzyme that is suppressed by 5-fluoro-deoxyuridine-monophosphate (FdUMP), an active metabolite of fluorouracil [9]. FdUMP and TS form covalent ternary complexes with 5, 10-methylene-tetrahydrofolate that subsequently inhibit DNA synthases [9,10]. Colorectal cancer with both low DPD and low TS mRNA expressions has been reported to show greater antitumor effects in 5-FU-based chemotherapy [11,12]. Fluorouracil is converted to active metabolites by phosphorylation through three different pathways, and TP and OPRT are the key enzymes in two of these pathways [13]. Thymidine phosphorylase is an enzyme that activates 5'-deoxy-5-fluorouridine to 5-FU and then 5-FU to 5-fluoro-2'-deoxyuridine. Orotate phosphoribosyltransferase is an enzyme that converts 5-FU to 5-fluorouridine-5'-monophosphate (FUMP) and is considered to predominantly inhibit RNA synthesis. High expression of TP in a tumor is correlated with a high response rate to 5'-deoxy-5-fluorouridine [14,15] and high expression of OPRT in a tumor is correlated with sensitivity to 5-FU [13,16]. For these reasons, many studies have reported that the activities of these enzymes have been associated with sensitivity to 5-FU-based chemotherapy in gastric cancer [17-19] and colorectal cancer [11,12,20-23].

These observations were based on gene expressions evaluated by using fresh frozen materials, which were composed of cancer cells, stromal cells in the cancer tissues, and even normal tissues. Because gastric and breast cancers contain large amounts of stromal cells in the cancer tissues, gene expression evaluated by ordinary methods reflects that of the cancer tissues, but not cancer cells. To evaluate gene expression of the cancer cells alone, it is essential to isolate the cancer cells from the stromal cells. To achieve this, we used a laser capture microdissection plus the real time reverse transcription-polymerase chain reaction (RT-PCR) method (LCM+RT-PCR) on formalin-fixed paraffin-embedded (FFPE) samples. In our previous report, in which TS, DPD, and TP gene expressions in breast cancers were evaluated by this method, we disclosed that the gene expressions in cancer cells were significantly different from those in stromal cells [24]. Gastric cancer, which contains large amounts of stromal cells in the cancer tissues, may show different gene expression between cancer cells and stromal cells as in breast cancers. In this study, gene expression levels of TS, DPD, TP, and OPRT in cancer cells of gastric cancer tissues were separately quantified from those in stromal cells by using the LCM+RT-PCR method. We also investigated those genes in colorectal cancer, which contains small amounts of stromal cells.

## Methods

### Patient and samples

Formalin-fixed paraffin-embedded samples from 47 patients with gastric cancers and 43 with colorectal cancers who underwent surgery were studied. This study was approved by the Institutional Review Board of the Tokyo Medical Dental University and all patients gave written consent.

### Laser capture microdissection

A representative FFPE tumor was selected by a pathologist after examination of the hematoxylin and eosin-stained slides. 10 μm thick sections were stained with nuclear fast red to enable visualization of histology for LCM (P.A.L.M. Microlaser Technologies AG, Munich, Germany). Caner cells (Ca), cancerous stroma (Str) of the sample were dissected using the LCM technique.

**Figure 1:**
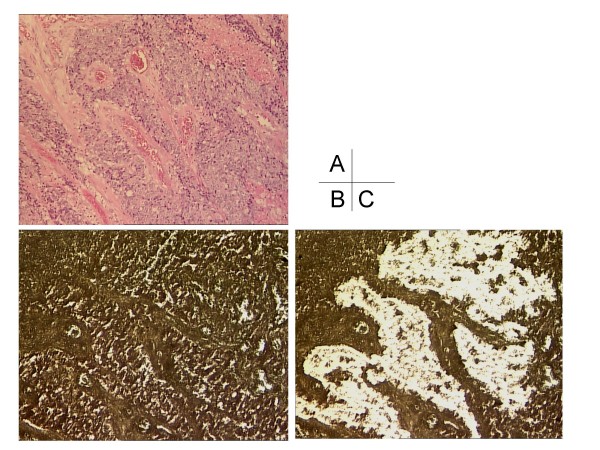
**1A: Formalin-fixed, paraffin-embedded samples were stained with H & E.** 1B: Formalin-fixed, paraffin-embedded samples were stained with nuclear fast red. 1C: By laser capture microdissection , only tumor cells were dissected.

Formalin-fixed, paraffin-embedded tumor specimens and adjacent normal tissues were cut into serial sections with a thickness of 10 μm. For the pathological diagnosis, one slide was stained with H&E and evaluated by pathologist (Figure 1A). Other sections were stained with nuclear fast red (NFR, American MasterTech Scientific Inc., Lodi, CA) to enable visualization of histology (Figure 1B). Laser capture microdissection (P.A.L.M. Microlaser Technologies AG, Munich, Germany) was performed in all the tumor samples to ensure that only tumor cells were dissected (Figure 1C). At least 25 mm^2 ^of tumor tissue or stromal tissue are collected from each FFPE block. Depending on the percentage of tumor tissue in the specimen, this generally can require from one to four 10 μm sections.

### Real-time quantitative RT-PCR

RNA was isolated from these using a novel, proprietary procedure (Response Genetics, Los Angels, CA: United States Patent Number 6,248,535). After RNA isolation, cDNA was derived from each sample according to a previously described procedure [25].

Target cDNA sequence were amplified by quantitative PCR using a fluorescence-based real-time detection method(ABI PRISM 7900 Sequence Detection System [Taqman]; Applied Biosystems, Foster City, CA)as previously described [26,27]. The 25 μl PCR reaction mixture contained 600 nmol/l of each primer (Table 1), 200 nmol/l each of dATP, dCTP, and 1× Taqman buffer A containing a reference dye (all reagents were supplied by Applied Biosystems, Foster City, CA). The PCR conditions were 50°C for 10 s and 95°C for 10 min, followed by 42 cycles at 95°C for 15 s and 60°C for 1 min. TS, DPD, TP and OPRT gene expressions in each part of the tumors were quantified as ratios between two absolute measurements (gene of interest/beta-actin).

**Table 1:** Polymerase chain reaction primers and probes

Primers/probes	Sequence
TS2-764F(18 bp)	GCCTCGGTGTGCCTTTCA
TS2-830R(17 bp)	CCCGTGATGTGCGCAAT
Probe TS2-785T(21 bp)	6FAM-TCGCCAGC-
DPD3a-51F(19 bp)	AGGACGCAAGGAGGGTTTG
DPD3a-134R(20 bp)	GTCCGCCGAGTCCTTACTGA
Probe DPD3a-71T(29 bp)	6FAM-CAGTGCCTACAGTCTC-
TP3-700F(17 bp)	CCTGCGGACGGAATCCT
TP3-770ZR(20 bp)	GCTGTGATGAGTGGCAGGCT
Probe TP3-722T(25 bp)	6FAM-CAGCCAGAGATGTGA-
β-actin-592F(18 bp)	TGAGCGCGGCTACAGCTT
β-actin-651R(22 bp)	TCCTTAATGTCACGCACGATTT
Probe β-actin-611T(18 bp)	6FAM-ACCACCACGGCCGAGCGG

bp, base pairs

### Statistical analysis

Comparison of mRNA levels between matched cancer cells and cancerous stroma was made with the Wilcoxon's rank test.

## Results

### Gene expressions in cancer cells (Ca) and cancerous stroma (Str) in gastric cancer

**Figure 2:**
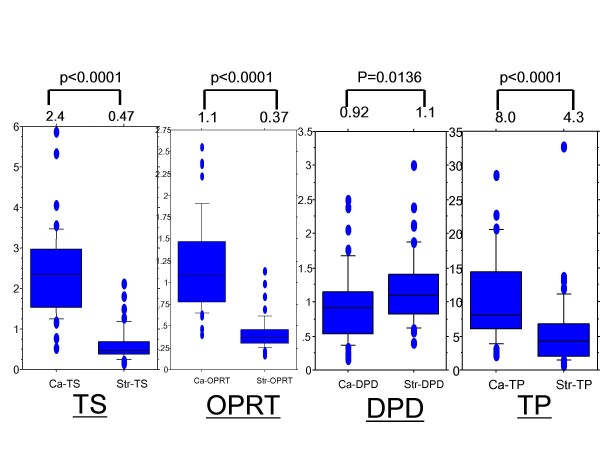
**Gene expressions in cancer cells and cancerous stroma in gastric cancer**. Expression levels of thymidylate synthase, orotate phosphoribosyltransferase, dihydropyrimidine dehydrogenase, and thymidine phosphorylase mRNA in cancer cells (Ca) and cancerous stroma (Str) were evaluated by laser capture microdissection plus reverse transcription-polymerase chain reaction. Thymidylate synthase, orotate phosphoribosyltransferase, and thymidine phosphorylase expression levels were higher in Ca than in Str. The dihydropyrimidine dehydrogenase expression level was higher in Str than in Ca.

As shown in Figure 2, the median TS mRNA level was 2.4 in Ca and 0.47 in Str. The TS gene expression levels were significantly higher in Ca than in Str (p < 0.0001). The median DPD mRNA level was 0.92 in Ca and 1.1 in Str. The DPD gene expression levels were significantly lower in Ca than in Str (p = 0.0136). The median TP mRNA level was 8.0 in Ca and 4.3 in Str. The TP gene expression levels were significantly higher in Ca than in Str (p < 0.0001). The median OPRT mRNA level was 1.1 in Ca and 0.37 in Str. The OPRT gene expression levels were significantly higher in Ca than in Str (p < 0.0001).

### Gene expressions in cancer cells (Ca) and cancerous stroma (Str) in colon cancer

**Figure 3:**
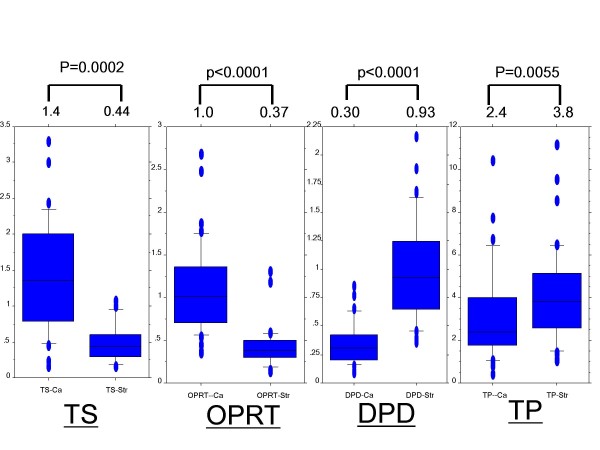
**Gene expressions in cancer cells and cancerous stroma in colon cancer**. Expression levels of thymidylate synthase, orotate phosphoribosyltransferase, dihydropyrimidine dehydrogenase, and thymidine phosphorylase mRNA in cancer cells (Ca) and cancerous stroma (Str)were evaluated by laser capture microdissection plus reverse transcription-polymerase chain reaction. Thymidylate synthase and orotate phosphoribosyltransferase expression levels were higher in Ca than in Str. Dihydropyrimidine dehydrogenase and thymidine phosphorylase expression levels were higher in Str than in Ca.

As shown in Figure 3, the median TS mRNA level was 1.4 in Ca and 0.44 in Str. The TS gene expression levels were significantly higher in Ca than in Str (p = 0.0002). The median DPD mRNA level was 0.30 in Ca and 0.93 in Str. The DPD gene expression levels were significantly lower in Ca than in Str (p < 0.0001). The median TP mRNA was 2.4 in Ca and 3.8 in Str. The TP gene expression levels were significantly lower in Ca than in Str (p = 0.0055). The median OPRT mRNA level was 1.0 in Ca and 0.37 in Str. The OPRT gene expression levels were significantly higher in Ca than in Str (p < 0.0001).

## Discussion

In this study, all the gene expressions were successfully estimated separately in cancer cells and cancerous stroma of human gastric and colon cancers using the LCM+RT-PCR technique. All of them showed significantly different expression levels between cancer cells and cancerous stroma.

In both gastric and colon cancers, higher gene expression levels of TS and OPRT were observed in cancer cells than in cancerous stroma and a lower gene expression level of DPD was observed in cancer cells than in cancerous stroma. These gene expressions might be controlled by a common regulatory mechanism in gastric and colon cancers. In our previous study, these genes showed the same tendency in breast cancer [24]. TS is the enzyme for DNA synthesis and cell proliferation and because cancer cells grow more rapidly than normal cells, TS gene expression is thought to be up-regulated in cancer cells. Several studies have revealed that the expression of OPRT is increased in several types of carcinoma, including gastric and colorectal carcinomas [28-30]. OPRT is a nucleotide metabolic enzyme that is essential for cell proliferation. Thus, OPRT gene expression is thought to be up-regulated in cancer cells, like TS gene expression [31,32]. Evaluations of comparisons of the expression levels of DPD in cancer cells and in normal tissues are controversial. Differing results might be affected by the use of tumor tissue samples with various amounts of stromal tissue. However, in the recent study, the amount of stromal cells was taken into consideration, and the results agreed with those of previous reports that DPD expression in cancer cells was lower than in normal tissues and in stromal cells [33,34]. McLead *et al. *reported that the down-regulation of DPD expression may create a favorable environment for tumor growth. Low expression levels of DPD and decreased catabolism of uracil in cancer cells suggest that pyrimidine nucleotide pools increase [35]. On the other hand, regarding the gene expression level of TP, the opposite result was observed. Although the gene expression level of TP was higher in cancer cells than in cancerous stroma in gastric cancer, it was lower in cancer cells than in cancerous stroma in colon cancer. It has been reported that certain solid tumors, including gastric and breast cancers, expressed elevated levels of TP as compared with stromal tissues [36-39]. In colon cancer, several immunohistochemical studies reported that cancer cells had TP expression [39,40], while other studies reported that most cells expressing TP were stromal cells, especially macrophages and lymphocytes [41,42]. It was shown by analyzing the expression level of TP in cancer cells and cancerous stroma separately that cancer cells had TP expression, although stromal cells had higher TP expression than cancer cells. These current results regarding the expression of TS, DPD, OPRT, and TP agree with the previous data.

In previous studies, biochemical assays, immunohistochemistry, enzyme-linked immunosorbent assay (ELISA), and reverse transcription-polymerase chain reaction (RT-PCR) have been used to evaluate the protein expression related to enzymes catabolizing 5-FU. Biochemical assays are often impossible to perform with minimal clinical samples. Many institutions have stored tissue samples as paraffin-embedded specimens after surgical resection, so immunohistochemical assays are convenient and inexpensive when performed on paraffin-embedded specimens. However, this method does not yield quantified results. Amplification by PCR may be performed on fresh frozen samples from resected cancer specimens to study gene expressions. However, the results are dependent on how promptly the samples were collected and stored. This method is not practical for the purpose of retrospective studies. In fresh frozen samples of cancer tissues, contamination with cancerous stroma and even normal tissue cannot be avoided. It is known that gastric and breast cancers contain large amounts of stromal tissue. We thought that the contaminations might influence the results of PCR, so we adopted LCM+RT-PCR. Laser capture microdissection provides selective isolation of defined cell populations from heterogeneous tissue sections [43]. Moreover, the availability of real-time RT-PCR technology combined with the extraction of RNA from paraffin-embedded specimens allows quantitative and accurate measurement of gene expressions [25,44]. This novel method made it possible to analyze only cancer cells, so in spite of amounts of cancerous stroma, we could analyze intratumoral gene expressions equally. Furthermore, this method has another advantage. Because cancer cells and cancerous stroma are extracted separately within the same paraffin section, we can perform the analysis from a small amount of resected specimen.

The time when various chemotherapies, including monoclonal antibody therapy has come, but the chemotherapy with 5-FU-based regimen has still played an important role as the treatment for many cancers. Accordingly it is thought to be useful to estimate gene expressions of enzymes, that are related to catabolism of 5-FU, in Ca and Str separately by LCM+RT-PCR. Though we need further investigate the relationship between those gene expressions and 5-FU sensitivity in clinical setting, we believe that with this technique it may be possible to predict the sensitivity of the agents before treatment using a small amount of a biopsy specimen and it leads to establish a tailor-made treatment for cancer patients.

## Conclusion

In this study, by using LCM+RT-PCR, we could analyze gene expressions in cancer cells and stromal tissues separately in FFPE gastric and colon cancer specimens. This method may make it possible to accurately analyze gene expressions in cancer cells from a small amount of a biopsy specimen in spite of the amount of stromal tissue and intratumoral heterogeneity.

## Competing interests

The authors declare that they have no competing interests.

## Authors' contributions

HM prepared the formalin-fixed paraffin-embedded (FFPE) samples and drafted the manuscript. KD and PVD performed the LCM+RT-PCR. HU performed the statistical analysis. KS oversaw this study. All authors read and approved the final manuscript.

## Pre-publication history

The pre-publication history for this paper can be accessed here:


